# Exploring the Contribution of Two Phosphorus-Based Groups to Polymer Flammability via Pyrolysis–Combustion Flow Calorimetry

**DOI:** 10.3390/ma12182961

**Published:** 2019-09-12

**Authors:** Rodolphe Sonnier, Belkacem Otazaghine, Christelle Vagner, Frédéric Bier, Jean-Luc Six, Alain Durand, Henri Vahabi

**Affiliations:** 1 Centre des Matériaux des Mines d’Alès (C2MA), IMT Mines Alès-6, Avenue de Clavières, CEDEX 30319 Alès, France; belkacem.otazaghine@mines-ales.fr; 2 Laboratoire Matériaux Optiques, Photonique et Systèmes (LMOPS), CentraleSupélec, Université de Lorraine, F-57000 Metz, France; christelle.vagner@univ-lorraine.fr (C.V.); henri.vahabi@univ-lorraine.fr (H.V.); 3 Laboratoire Matériaux Optiques, Photoniques et Systèmes, CentraleSupélec, Université Paris-Saclay, 57070 Metz, France; 4 Aix Marseille Univ, French National Center for Scientific Research (CNRS), MADIREL UMR 7246, F-13397 Marseille, France; 5 Laboratoire de Chimie Physique Macromoléculaire (LCPM), Université de Lorraine, UMR7375, F-54001 Nancy, France; frederic.bier@gmail.com (F.B.); jean-luc.six@univ-lorraine.fr (J.-L.S.); alain.durand@univ-lorraine.fr (A.D.); 6 Laboratoire de Chimie Physique Macromoléculaire (LCPM), French National Center for Scientific Research (CNRS), UMR7375, F-54001 Nancy, France

**Keywords:** polymer flammability, van Krevelen approach, group contributions, pyrolysis–combustion flow calorimetry, phosphorus-containing flame retardant

## Abstract

From a set of around 100 phosphorus-containing polymers tested in pyrolysis–combustion flow calorimetry, the contributions to flammability of two phosphorus-containing pendant groups (called 9,10-dihydro-9-oxa-10-phosphaphenanthrene-10-oxide (DOPO) and PO_3_) were calculated using an advanced method previously proposed and validated. The flammability properties include total heat release (THR) and heat release capacity (HRC) measured in standard conditions, i.e., anaerobic pyrolysis and complete combustion. The calculated contributions are in good agreement with the main modes of action of both phosphorus groups, i.e., flame inhibition for DOPO and char promotion for PO_3_. Moreover, the results provide first conclusions about the cooperative interaction between phosphorus and nitrogen, as well as the influence of the architecture of tested co-polymers.

## 1. Introduction

Phosphorus is currently considered as a key element to develop flame-retardant (FR) materials. Indeed, it can act as a char promoter in the condensed phase and/or as a flame inhibitor in the gaseous phase [1]. Numerous phosphorus compounds are currently available, mainly as additives for polymers. Nevertheless, phosphorus groups can also be chemically incorporated into thermoplastic chains or thermoset networks [2,3]. This so-called reactive approach is expected to enhance the durability of materials by preventing the migration of FR outside the polymer matrix [4]. Moreover, the transparency, as well as the mechanical properties, may be more easily maintained through the reactive approach, since phosphorus additives generally behave as plasticizers and lower the glass transition temperature of final materials compared to that of bulk polymers. Even if the phosphorus-containing groups chemically bonded to polymers can also affect the glass transition temperature, this effect may be significantly reduced in comparison with phosphorus additives. Finally, several studies suggested that the reactive approach was more efficient than the additive one [5,6,7,8,9]. A review paper about the flame retardancy of phosphorus-containing polymers was recently published [10].

The efficiency of phosphorus, as well as its mode of action in the condensed or gaseous phase, depends on a couple of parameters, including its oxidation state [11,12,13,14] and the host polymer [15]. When phosphorus is covalently bonded to the polymer, its exact position on the main chain also has an effect. It can be located in the backbone as in polyphosphazenes, which exhibit high flame retardancy [16]. Nevertheless, most often, phosphorus groups are positioned as pendant groups. Thus, selecting the exact chemical structure of a phosphorus-containing monomer may be an efficient way to increase the FR efficiency. Additionally “synergism” with other chemical groups (aromatic rings, as well as “flame-retardant elements” such as nitrogen, sulfur, etc.) is also postulated, and many attempts were made to prepare flame-retardant polymers combining phosphorus and nitrogen [17,18], sulfur [19,20], silicon [21,22], and bromine [23], as well as calcium or zinc [24,25,26]. Nevertheless, “synergism” is most often difficult to confirm.

Among the phosphorus-containing groups incorporated into polymers, 9,10-dihydro-9-oxa-10-phosphaphenanthrene-10-oxide (DOPO), as well as its derivatives [8,27,28,29,30,31], and phosphonate groups [9,17,32,33,34,35,36,37] are probably the most widely studied. DOPO mainly acts as a flame inhibitor, while phosphonate groups promote charring with no or little effect in the gaseous phase. In most cases, these groups were added as pendant groups even if some attempts were carried out to incorporate phosphonate groups within the polymer backbone [32,33].

Predicting the flammability of polymers was recently successful on the basis of molecular structure of repeat units using experimental data obtained by pyrolysis–combustion flow calorimetry (PCFC) and considering a simplistic model based on the additivity of molar groups contributions [38,39]. This approach was originally proposed by van Krevelen [40] and was firstly applied to PCFC data by Lyon et al. [41,42]. A database gathering the contributions for about 45 chemical groups was proposed and validated for about 140 thermoplastics and thermosets [38,39]. Such a model should help chemists to consider polymeric structures to be synthetized in order to obtain low flammability without proceeding through a long and expensive trial-and-error process. Moreover, it should help to identify interesting structures. Indeed, interactions between chemical groups may lead to a discrepancy between the experimental and calculated flammability properties of specific polymers, while the model is additive and does not consider contributions from interactions.

Nevertheless, no satisfactory contribution was proposed for phosphorus-containing groups, mainly because too few phosphorus-containing polymers were tested [38,43]. Moreover, the phosphorus content in such polymers is usually low and, therefore, it is difficult to accurately calculate the contributions of phosphorus-containing groups to flammability.

In this work, the contributions of two phosphorus-containing pendant groups (namely, DOPO and PO_3_ groups) were calculated from a large set of around 100 polymers. These groups were chosen because they are widely used to impart flame retardancy to new polymers. Furthermore, they act according to different modes of action. Moreover, the content of both phosphorus-containing groups reaches high values in many polymers. The calculated contributions of both groups allow for reasonable fitting of the flammability properties of the polymers considered at the molecular scale. This approach also allows the formulation of preliminary conclusions about the interaction of two or more flame-retarding elements simultaneously present and about the architecture of co-polymers. To the best of the authors’ knowledge, this is the first time that the contributions of phosphorus-containing groups are satisfactorily calculated from such a large set of polymers.

## 2. Materials and Methods

Around 100 phosphorus-containing polymers grouped into eight series (from A to H) were studied (Table 1). For each series, the differences between the polymers were as follows: the nature of one or several co-monomers and/or the ratio between the co-monomers. Details about most of these polymers can be found elsewhere (references are given in the last column of Table 1). Their flammability was already reported in various articles except for polymers from series A and C.

The detailed synthesis procedure of polymers from series A is described in detail elsewhere [44]. The two-step synthesis strategy used the Atherton–Todd reaction [45,46]. The first step consisted of, firstly, a radical reaction which activated an alkenol (of variable length between 3 and 11 carbon atoms) in the presence of a radical initiator (azobisisobutyronitrile, AIBN) at 70 °C. The second step was the introduction of the methacrylic function and was realized by the nucleophilic substitution between the previous DOPO-alcohol and methacryloyl chloride. The DOPO-alkan-methacrylates were named DOPO-MnP, where n is the number of methylene groups contained in the aliphatic spacer.

Co-polymerization of the DOPO-containing monomer and methyl methacrylate was carried out in a test tube equipped with a three-way and a magnetic stirrer. The co-monomers and the initiator (AIBN, 1 wt.% compared to monomer) were solubilized in dimethyl sulfoxide (DMSO). Reaction time was fixed at 15 h and the temperature was 80 °C. Co-polymers were recovered by precipitation into diethyl ether. Finally, samples were dried 24 h in an oven at reduced pressure at 40 °C until constant weight.

**Figure 1:**
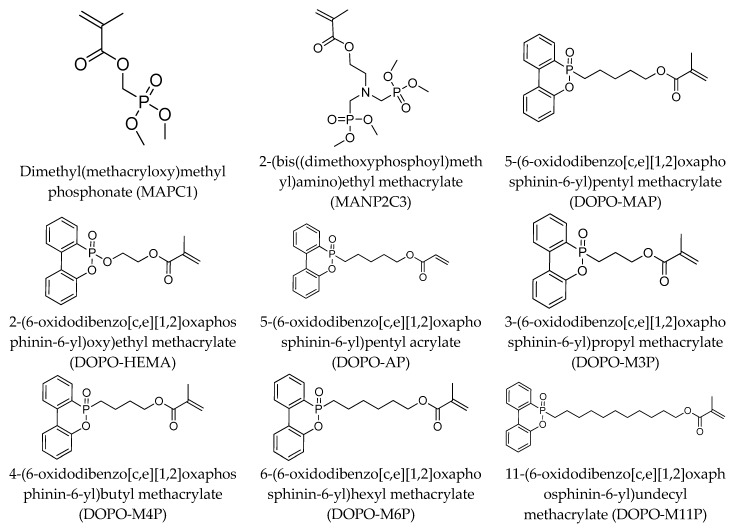
Structure of 9,10-dihydro-9-oxa-10-phosphaphenanthrene-10-oxide (DOPO)-functionalized monomers, MAPC1 and MANP2C3.

The structures of various phosphorus-functionalized monomers listed in Table 1 (series A, B, and C) are provided in Figure 1. Polymers from series A were statistical co-polymers of DOPO-containing monomers and methyl methacrylate (MMA) prepared using radical polymerization. DOPO-containing monomers were of acrylic (in one case) and mainly methacrylic type with DOPO as a pendant group. The final content of the phosphorus-containing monomer was up to 50% in moles. Homopolymers of DOPO-containing monomers were also prepared following the same procedure. Note also that the oxidation state of the phosphorus is not the same for all the co-polymers from series A (compare DOPO–2-(6-oxidodibenzo[c,e][1,2]oxaphosphinin-6-yl)oxy)ethyl methacrylate (HEMA) and others). Nevertheless, as shown below, the contributions calculated for this group allow predicting the flammability properties of all the co-polymers from series A, regardless of the true oxidation state of phosphorus.

Flammability of the polymers listed in Table 1 was analyzed using PCFC (from FTT, United Kingdom) under standard conditions, i.e., anaerobic pyrolysis from 25 to 750 °C at 1 °C/s in nitrogen and complete combustion in an excess of oxygen at 900 °C [48]. O_2_ and N_2_ volume fractions in the combustor were fixed at 0.2 and 0.8, respectively. The sample weight was typically 2–3 mg so that oxygen was never fully consumed. Moreover, the sample was considered as thermally thin.

The total heat release (THR) corresponds to the area under the heat release rate curve. The heat release capacity (HRC) generally corresponds to the peak of heat release rate (pHRR) divided by the heating rate. However, in some cases, several peaks can be observed. In such a case, the sum of the HRR peaks after deconvolution carried out using the FTT software is considered (sumHRC). When the different peaks do not overlap, the deconvolution is easy and unambiguous. For the cases where several peaks overlap, sumHRC was determined as previously described by summing the minimum number of Gaussian, Lorentzian, asymmetric Gaussian or Lorentzian, or asymmetric Gaussian–Lorentzian hybrid peaks needed to fit the HRR curve with an accuracy of at least 95% [41]. Obviously, the choice of the number of peaks influences the sumHRC.

Equations (1) and (2) explain how the total heat release (THR) and heat release capacity (HRC) of a given polymer can be calculated on the basis of the structure of its repeat unit.

(1) $$\mathrm{THR}=\;\underset{i}{\sum }w_{i}\times {\mathrm{THR}}_{i},$$

(2) $$\mathrm{HRC}=\;\underset{i}{\sum }w_{i}\times {\mathrm{HRC}}_{i},$$

where THR_i_ and HRC_i_ reflect the contributions of group i to THR and HRC, respectively, and w_i_ is the weight fraction of group i in the polymer.

Most of the polymers studied in this article have a low or negligible char yield (series A, for example). In some cases, these data were not recorded when PCFC analyses were carried out (series B, C, and F). Therefore, the char yield was not calculated and compared to experimental data. Nevertheless, the contribution to char (μ) of the new groups considered here can be calculated using Equation (3), from the comparison between the contribution to THR and the heat of complete combustion (Δh), as described in previous articles [38,39]. Walters et al. showed that the char composition is close to C_5_H_2_ in most cases [49]. The energy released by the complete pyrolysis and combustion of such char Δh_char_ is then 37.2 kJ/g. When the contribution to THR is significantly different from the heat of complete combustion, it means that contribution to char is significant. Δh is calculated using Huggett’s relation [50] considering the complete pyrolysis and combustion of the whole polymer structure.

Δh is calculated without considering the oxidation of nitrogen atoms since oxidation of nitrogen is believed to occur at much higher temperature [51].

(3) $$\mu =\frac{\Delta h-\mathrm{THR}}{{\Delta h}_{\mathrm{char}}}.$$

This method is simplistic because it considers that all the chars have the same composition. Nevertheless, the method allows for correct prediction of the THR and the char content of the polymers from only one parameter: the contribution to THR. This was the case in a previous work for a series of around 30 thermosets including high-charring polymers [39].

The calculation of the heat of complete combustion for phosphorus-containing groups requires information about the oxidation state of phosphorus after oxidation. Phosphorus was considered to be fully oxidized into P_2_O_5_, i.e., 2.5 atoms of oxygen for one atom of phosphorus. Lyon et al. proposed that phosphorus species were converted into H_3_PO_4_, i.e., 0.5P_2_O_5_ + 1.5H_2_O, which was similar to the calculation used in this work [52]. This leads to a slightly negative heat of complete combustion in the case of the PO_3_ group since this group is made up of three atoms of oxygen for one atom of phosphorus.

More details about the method to build the database of calculated group contributions step-by-step can be found in previous articles [38,39].

## 3. Results and Discussion

Some preliminary precisions about the model and its limitations are needed. Firstly, the model allows calculating negative contributions. From a physical point of view, negative contributions are meaningless. Indeed, one polymer containing only one group exhibiting negative contributions would present itself negative flammability properties. Nevertheless, if the content of the group having negative contributions is not too high, the flammability properties of the polymer remain positive. In that case, such a group may be considered as acting to its neighboring groups. It modifies their degradation pathway and reduces their contribution to flammability. When such an interaction is more or less systematic whichever the neighbors, it can be included into the own contributions of the group.

Of course, deviations between experimental and calculated values are unavoidable. A first reason is that the calculation of contributions evolves and depends on the set of studied polymers. However, the main reason is that the model is very simplistic. The decomposition pathway is never exactly as a sum of independent steps corresponding each one to a chemical group (which is the meaning of a model based on additive contributions). When the deviations are limited, it means that such a model remains a reasonable assumption. When the deviations are high, it can be assumed that interactions occur between some groups present in the polymer. In order to help to identify them, such interactions may be presented as another interest of the model.

The contributions of new groups calculated in this study are listed in Table 2. It should be noted that the contributions of trioxybenzene group (group 3 in Table 2) were already calculated in a previous work [39]. The contribution to THR found in the present work is the same but the contribution to HRC differs significantly (previous value was 100 J/g∙K and, in the present work, the calculations led to −350 J/g∙K). This discrepancy can easily be justified when considering that, in the previous article, the contribution was calculated using a series of only three polymers as compared to 29 in the present work. Thus, the previously proposed value of that contribution must be considered carefully. The new contribution appears more reliable because it allows for satisfactory prediction of the HRC of 29 polymers (series D and E), including the three phosphorus-free polymers used in the previous work.

Tentative contributions of the PO_3_ pendant group were also calculated in a previous work [38] but these values were reported as unsatisfactory. The new contributions shown in Table 2 allow for a more accurate prediction of the flammability properties of 57 polymers (including most of the polymers already studied).

Similarly, the contributions of methylene sulfide group (group 4 in Table 2, from series E) must be considered as tentative. Indeed, even if this group is present in 12 polymers, its weight fraction is reduced in all cases and, thus, its contribution has low influence on the predicted values. Therefore, we did not discuss these values in detail.

This illustrates that the database is still under construction, and some contributions can be modified in the future depending on the availability of new experimental data.

The contributions of DOPO were calculated from a large range of 35 homo- and co-polymers (series A). Its weight fraction ranged from 0.27 to 0.63. Therefore, its influence on the heat release may be very significant, which should be in favor of the accuracy of the estimated contributions.

**Figure 2:**
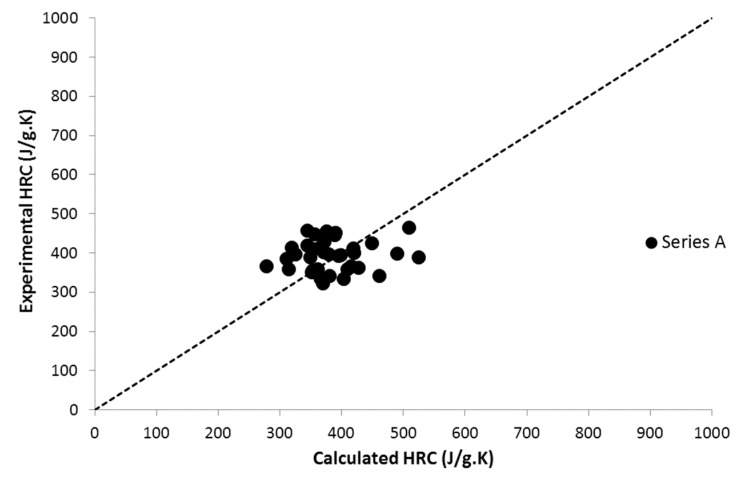
Experimental versus calculated heat release capacity (HRC) for 35 DOPO-functionalized polymers.

**Figure 3:**
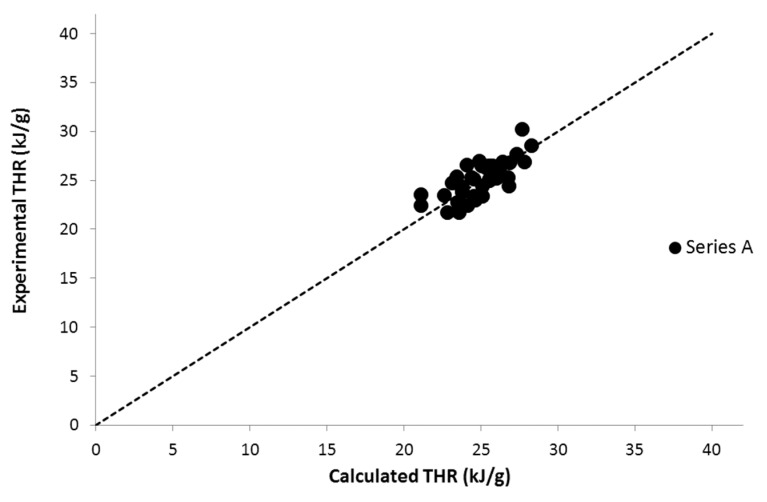
Experimental versus calculated total heat release (THR) for 35 DOPO-functionalized polymers.

Nevertheless, the THR and HRC of all these 35 polymers were in the same range (Figure 2 and Figure 3). HRC ranged from 300 to 500 J/g∙K and THR ranged from 20 to 30 kJ/g. This means that the contributions of DOPO are similar to the contributions of the other groups present in the polymers. Indeed, the contributions to HRC and THR are respectively 270 J/g∙K and 27 kJ/g, which are relatively high values. Therefore, DOPO cannot be considered as an efficient flame-retardant group considering the fact that values were measured in PCFC in standard conditions.

DOPO is well known to usually be a poor char promoter but efficient as a flame inhibitor. However, in standard conditions, combustion is complete. Indeed, the temperature of the combustor is 900 °C, while the combustion needs a temperature lower than 700 °C to be incomplete for most polymers. To the best of the authors’ knowledge, only one polymer, poly(4-bromostyrene), exhibited incomplete combustion at 900 °C [53,54]. Therefore, the efficiency of flame inhibitors as DOPO is underestimated when using these conditions, as already proven in a previous work [53]. As an example, 1 wt.% phosphorus provided by the incorporation of a DOPO-containing group significantly improved the flame retardancy of polyamide 11 according to limiting oxygen index (LOI) and UL94 tests even if flammability at microscale was not modified [31].

**Figure 4:**
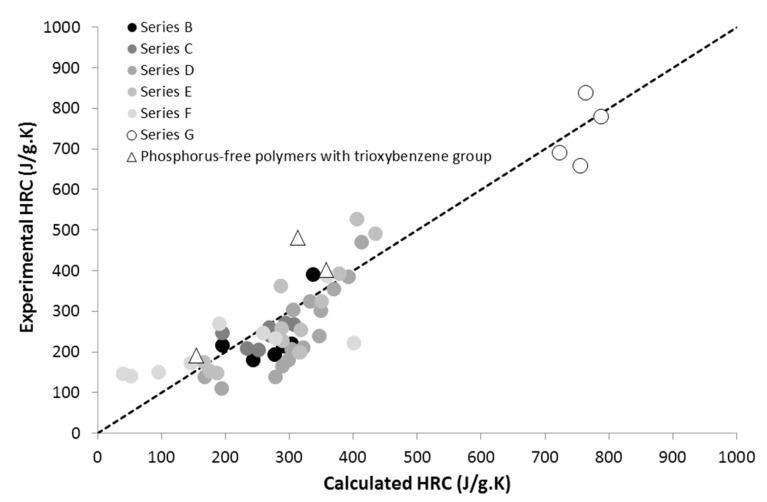
Experimental versus calculated HRC for polymers bearing PO_3_ pendant groups.

**Figure 5:**
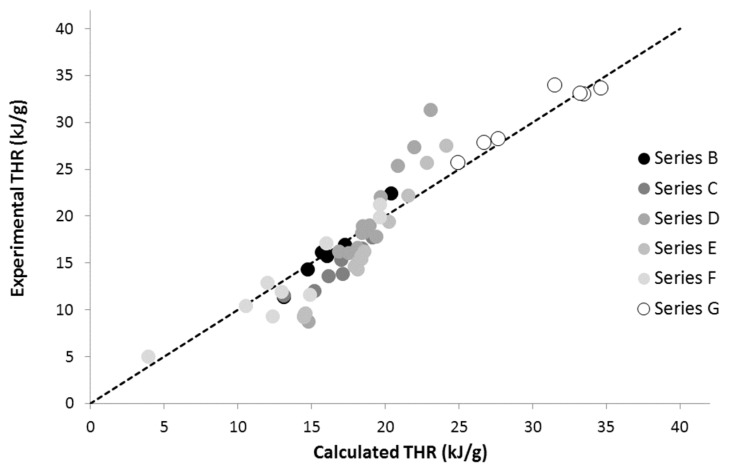
Experimental versus calculated THR for polymers bearing phosphonate pendant groups.

The calculated THR and HRC values for phosphonated polymers (series B, C, D, E, and F) are shown in Figure 4 and Figure 5. The weight fraction of the PO_3_ group changes greatly from one polymer to another, but it reaches up to 0.4 for homopolymer poly(MANP2C3). The agreement between calculated and experimental values can be considered as quite satisfactory. Some deviations from the dotted line are discussed below and may be attributed to the influence of the co-polymer architecture. In previous work, we also added three polymers for which the experimental HRC values were graphically obtained from the literature. These polymers were not considered in the present work.

It can be noted that the PO_3_ pendant group is much better than DOPO at improving flame retardancy. The PO_3_ pendant group has a significant effect on HRC, i.e., it reduces heat release rate to a great extent. Indeed, phosphonate is well known as a char promoter, modifying the decomposition mechanisms in the condensed phase (i.e., during the pyrolysis step). However, the contribution to THR is negative mainly because the PO_3_ group does not contain carbon atoms and its heat of combustion is low. Its contribution to char remains relatively limited (0.2 g/g). It is expected that this contribution would be much higher if this group was incorporated into the polymer backbone.

**Figure 6:**
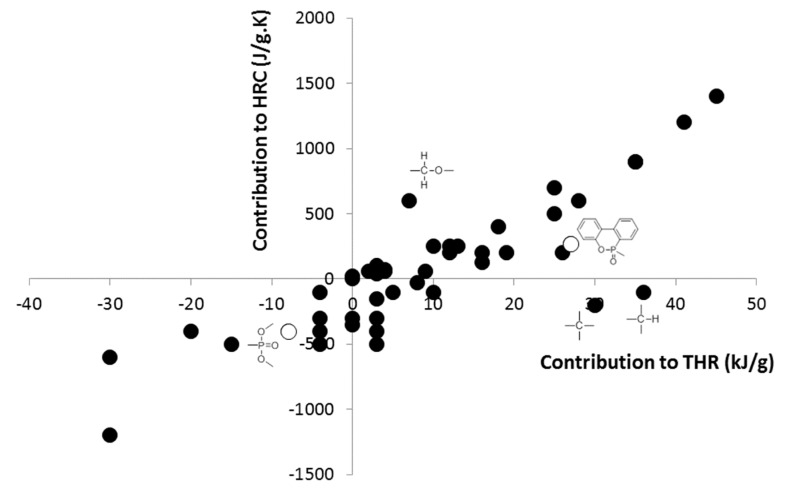
Contribution to HRC versus contribution to THR for 47 groups.

Figure 6 shows the contributions to HRC versus the contributions to THR for all groups already studied, i.e., 47 groups. The complete list of these groups and their corresponding contributions are available in previous papers [38,39]. Both DOPO and PO_3_ pendant groups follow the same rough tendency between these contributions. The higher the contribution to THR is, the higher the contribution to HRC is. This is not unexpected, but this rough tendency allows for identification of some exceptions, such as >C< and –CH< (high contribution to THR and low contribution to HRC) or >CH–O– (low contribution to THR and high contribution to HRC).

**Figure 7:**
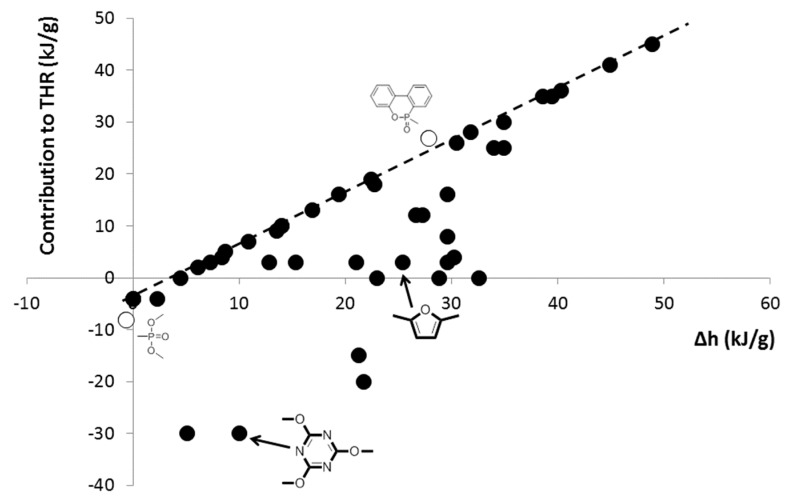
Contribution to THR versus Δh calculated from the Huggett relation for 47 groups.

Figure 7 shows the contribution to THR versus the heat of combustion calculated using Huggett’s relation. When both values are close, it means that the group does not significantly contribute to char. This is the case of DOPO. The PO_3_ pendant group contributes only moderately to char as already discussed, especially when comparing it with heteroaromatic groups (see some examples in Figure 7). Therefore, this data point is not far away from the dotted line.

### 3.1. Cooperative Interactions

As already mentioned, “synergism” between “flame-retardant” elements such as phosphorus, nitrogen, or sulfur is often claimed, but the evidence for cooperative interactions is rather scarce [55]. Moreover, it is unclear whether such interactions should occur only when both elements are directly linked through a covalent bond or even when both elements are not directly linked. In this work, several polymers containing N and P or S and P elements were studied but without direct bonding between these atoms. At least one carbon atom was present between N and P or S and P atoms in all corresponding polymers. In the case of sulfur-containing polymers, the contribution of CH_2_–S was not calculated independently (i.e., in phosphorus-free polymers); therefore, it is not possible to conclude anything.

**Figure 8:**
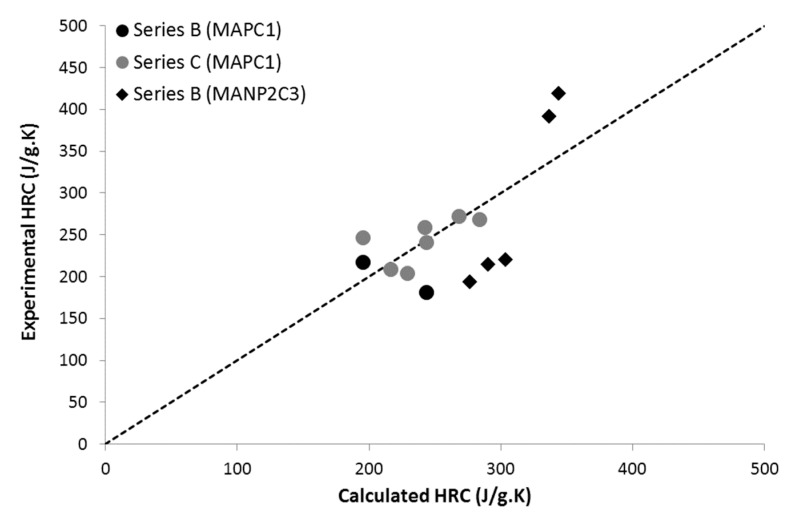
Experimental versus calculated HRC for polymers containing PO_3_ pendant groups from series B and C (i.e., methacrylate co-polymers including MAPC1 or MANC2P3 monomers).

The contributions of both –CH_2_–N< and PO_3_ pendant groups were calculated independently. It appears that these contributions also allow correctly predicting the flammability properties of the N- and P-containing polymers (containing MANP2C3 monomers, from series B) (Figure 8). Note that the weight fractions of the N- and P-containing groups are significant, especially for the homopolymer poly(MANP2C3). Therefore, it is assumed that no cooperative interaction occurs in these polymers when N and P atoms are not directly linked. Vahabi et al. also calculated the contributions of the PO_3_ group in MAPC1- and MANC2P3-containing co-polymers using another approach [35]. They also concluded that the contributions to effective heat of combustion (EHC) and char yield were similar in both co-polymers (i.e., the contribution to THR was also similar). Nevertheless, the contributions to HRC were −258 J/g∙K and −549 J/g∙K for PO_3_ groups in MAPC1-containing co-polymers and in MANC2P3-containing co-polymers, respectively. However, the fitting of calculated values with experimental ones was less satisfying, especially for MAPC1-containing co-polymers. It is noteworthy that these values are quite close to the contribution calculated in the present work (−400 J/g∙K).

This first example illustrates one main advantage of the present database based on the Van Krevelen method. Indeed, a cooperative effect can be highlighted or rejected on the basis of a quantitative assessment.

**Figure 9:**
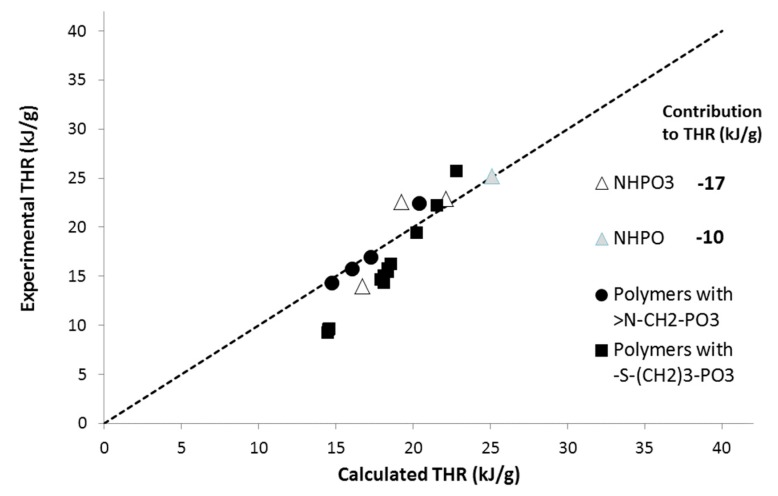
Experimental versus calculated THR for various polymers (from series B, E, and H).

Previously, Dumitrascu and Howell synthetized and analyzed other polymers containing phosphorus groups including covalent bonds between N and P [17]. Nevertheless, the number of polymers was too limited. Moreover, the article provides only THR but not sumHRC values. Therefore, these polymers were not plotted in the previous figures (series H). Nevertheless, we calculated the contribution of these groups to THR to properly fit the experimental THR. The correlations between experimental and calculated THR, as well as the corresponding contributions to THR, are shown in Figure 9. Data for other polymers containing N and P or S and P atoms (without direct bonding—from series B and E) were also added.

To correctly fit the experimental THR, the contributions of NHPO_3_ and NHPO would be close to −17 and −10 kJ/g, respectively. These values are very low and correspond to contributions to char close to 0.45. Indeed, the residue contents for these polymers are significantly higher than for their counterparts containing only phosphorus groups. Based on these preliminary results, it seems that a high flame-retardant effect may be expected from groups containing an N–P bond.

### 3.2. Influence of the Detailed Structure of Co-Polymers

Co-polymers of MMA and MAPC1 were synthetized by Vahabi et al. and Canniccioni et al. [35,47]. The former prepared random co-polymers while the latter synthesized block co-polymers. Moreover, we also studied physical blends of poly(MMA) (PMMA) and poly(MAPC1) (PMAPC1). The THR of all these materials can be predicted accurately using the contributions to THR previously calculated. In other words, there is no difference in THR values between random and block co-polymers or physical blends if the weight fractions of MMA and MAPC1 are the same.

**Figure 10:**
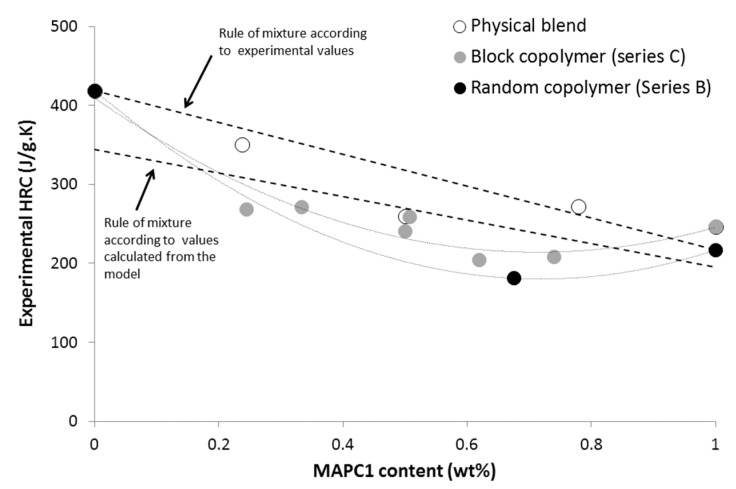
Experimental HRC versus MAPC1 content for various polymers (from series B and C).

Prediction of HRC is also rather reasonable but not perfect for block and random co-polymers (Figure 4). Figure 10 plots the experimental HRC versus MAPC1 content in the co-polymers as in physical blends in order to highlight possible differences between these materials. A slightly negative deviation is highlighted for co-polymers, especially the random co-polymer. For a similar composition, HRC is the highest for physical blend, and the lowest for co-polymers. Nevertheless, this deviation appears quite limited and cannot be considered as significant on the basis of experimental uncertainties. Further investigations are needed to draw clear conclusions on that point.

Interestingly, the lowest value for series B and C (i.e., random and block co-polymers) was not obtained for the homopolymer PMAPC1 but for an MAPC1 weight fraction close to 0.6–0.7. This fraction corresponds to a ratio between the acrylate COO group and PO_3_ group close to 2. This ratio is exactly the same as the one obtained in a previous work on phosphorus-containing oligomers and polymers [43]. Indeed, in this previous work, a method was presented to assess the interactions between groups based on PCFC results from another set of molecules and macromolecules. Cooperative interaction was the highest for this ratio between ester and PO_3_ groups.

### 3.3. Comparison of the Contributions Calculated from Other Works

**Figure 11:**
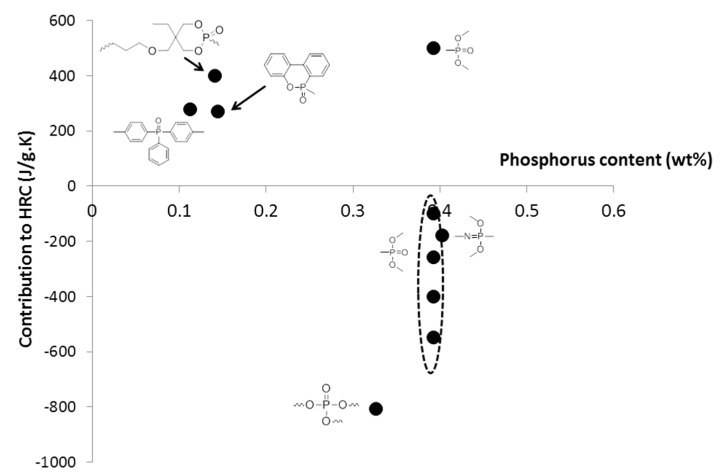
Contributions to HRC for various phosphorus-containing groups according to different works.

**Figure 12:**
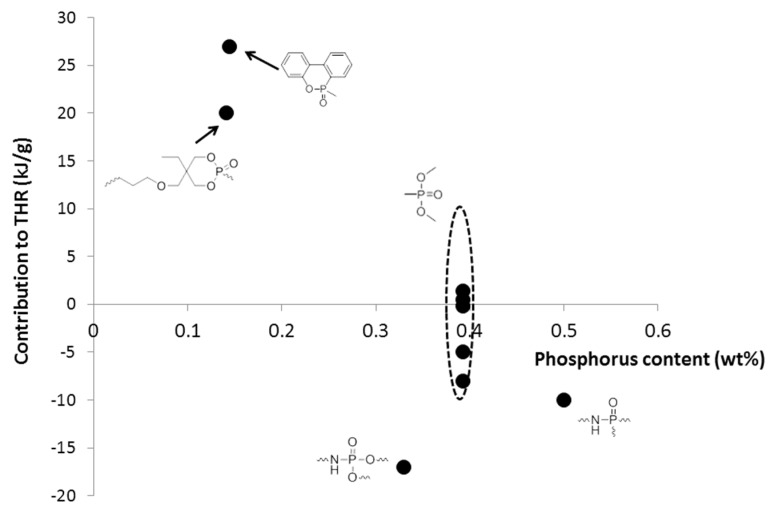
Contributions to THR for various phosphorus-containing groups according to different works.

It is interesting to compare the calculated contributions in the present work to those already proposed (Table 3). The comparison must be considered carefully because different approaches were followed according to the references. Moreover, some contributions were calculated with a very limited number of polymers (for example, in this work, NHPO and NHPO_3_). It is noteworthy that the contributions to THR and HRC of phosphorus groups without carbon atoms can be slightly positive or negative, confirming the flame-retardant effect of phosphorus (Figure 11 and Figure 12). The only exception is the contribution to HRC of the PO_3_ group calculated from a preliminary work [43]. This may be due to the small size of some molecules. The decomposition pathway of such molecules may be different from that of polymers.

**Figure 13:**
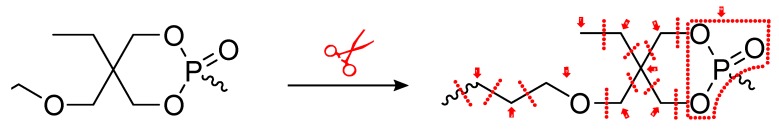
Structure fragmentation of dioxaphosphorinane group into smaller groups.

When the phosphorus group contains a significant carbon content, its contributions are notably higher (Figure 11 and Figure 12). The effect of phosphorus is then “diluted” and is hardly worthy of being highlighted. Nevertheless, it is noteworthy that a non-aromatic heterocycle can be decomposed into the smallest groups in the present model. Therefore, the contributions of the dioxaphosphorinane group studied in a previous work [43] can also be calculated from the contributions of small groups including the PO_3_ group (see Figure 13 for the structure fragmentation of a dioxaphosphorinane group). When using these contributions calculated from the present work (given in Table 2 for the PO_3_ group) and previous works (shown in Reference [38] for other groups), the contributions to THR and HRC of dioxaphosphorinane are 18.9 kJ/g and 433 J/g∙K, respectively. This is in very good agreement with the contributions calculated previously: 20 kJ/g and 400 J/g∙K considering the dioxaphosphorinane as a whole.

## 4. Conclusions

This attempt to calculate the contributions to flammability of phosphorus groups is the first considering nearly 100 polymers. Taking into account the increasing interest of phosphorus-containing FR compounds, such correlations become highly suitable. The calculated contributions are in good agreement with the main mode of action of DOPO and PO_3_ groups. DOPO is mainly a flame inhibitor and its contributions are high when combustion is complete as in standard PCFC conditions. On the contrary, the PO_3_ pendant group is a char promoter, modifying the decomposition rate. Its contribution to HRC is very low, evidencing its flame-retardant effect. Its contribution to char remains limited.

The model allows calculating the flammability properties according to the additivity of molar contributions without considering any interactions between groups. Therefore, the deviation between predicted and experimental values highlights a possible cooperative or antagonistic effect. First conclusions can be drawn on the basis of the present results. The combination of N and P atoms does not act cooperatively when these atoms are not directly bonded. The architecture of co-polymers (random versus block co-polymers) has no or limited effect on flammability. Interactions between ester and PO_3_ groups may be beneficial to reduce flammability. Even if these conclusions must be considered with caution and require further investigations, these examples illustrate the usefulness of the model and the related database.

## Figures and Tables

**Table 1:** List of polymers tested in the present study. DOPO—9,10-dihydro-9-oxa-10-phosphaphenanthrene-10-oxide; MAPC1—dimethyl(methacryloxy)methyl phosphonate; MANP2C3—2-(bis((dimethoxyphosphoyl)methyl)amino)ethyl methacrylate.

Series	Description	Number of Tested Polymers	Structure and Content (wt.%) of the Phosphorus Group	Reference
**A**	DOPO-containing acrylate and methacrylate co-polymers	35	DOPO Up to 63 wt.%	This work
**B**	MAPC1 and MANP2C3-containing methacrylate random co-polymers	6	PO_3_ Up to 41 wt.%	[35]
**C**	MAPC1-containing methacrylate block co-polymers	7	PO_3_ Up to 38 wt.%	[47]
**D**	Phosphonate-containing epoxy thermosets (including trioxybenzene group **)	17	PO_3_ Up to 14 wt.%	[36]
**E**	Phosphonate and sulfur-containing epoxy thermosets (including trioxybenzene and methylene sulfide groups **)	12	PO_3_ Up to 8 wt.%	[9]
**F**	Phosphonate-containing co-polymers	9	PO_3_ Up to 60 wt.%	[38]
**G**	Polymers from phosphorus-modified styrene monomers	7	PO_3_ Up to 35 wt.%	[17,37]
**H** *	Polymers from phosphorus-modified styrene monomers	4	NHPO and NHPO_3_ Up to 39 wt.%	[17]

* Only total heat release (THR) values were considered in the present article; ** See Table 2.

**Table 2:** List of groups studied in the present work and their estimated contributions to flammability. HRC—heat release capacity.

Group	Number of Polymers	Molar Mass (g/mol)	Contribution to			
			THR (kJ/g)	HRC (J/g∙K)	Δh (kJ/g)	Char (g/g)
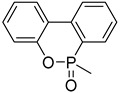	35	215	27	270	27.8	0.02
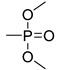	57	79	-8	−400	-0.7	0.20
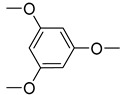	29	123	0	−350	23	0.62
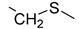	12	46	5	−300	22.8	0.48

**Table 3:** List of phosphorus-containing groups and the corresponding contributions to flammability.

	Contribution to THR (kJ/g)	Contribution to HRC (J/g∙K)	Reference
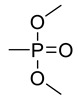	−8	−400	This work
	−5	−100	[38]
	−0.2 *	−258	[35]
	1.4 *	−549	[35]
	0.5	500	[43]
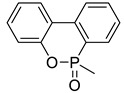	27	270	This work
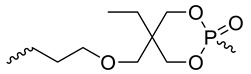	20	400	[43]
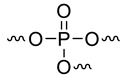	/	-807	[42]
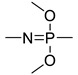	/	−179	[42]
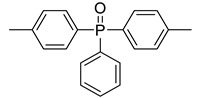	/	279	[42]
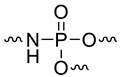	−17	/	This work
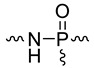	−10	/	This work

* Calculated from contributions to effective heat of combustion (EHC) and char.
